# The genome sequence of the millipede, *Choneiulus palmatus* (Nĕmec, 1895) (Julida: Blaniulidae)

**DOI:** 10.12688/wellcomeopenres.24885.1

**Published:** 2025-09-15

**Authors:** Steve J. Gregory, Gregory D. Edgecombe, Natural History Museum Genome Acquisition Lab, Darwin Tree of Life Barcoding Collective, Wellcome Sanger Institute Tree of Life Management, Samples and Laboratory team, Wellcome Sanger Institute Scientific Operations: Sequencing Operations, Wellcome Sanger Institute Tree of Life Core Informatics team, Tree of Life Core Informatics collective, Darwin Tree of Life Consortium

**Affiliations:** 1 British Myriapod & Isopod Group, Oxford, England, UK; 2 Natural History Museum, London, England, UK

**Keywords:** Choneiulus palmatus; Millipede; genome sequence; chromosomal; Julida

## Abstract

We present a genome assembly from an adult female *Choneiulus palmatus* (Millipede; Arthropoda; Diplopoda; Julida; Blaniulidae). The genome sequence has a total length of 626.52 megabases. Most of the assembly (92.77%) is scaffolded into 14 chromosomal pseudomolecules. The mitochondrial genome has also been assembled, with lengths of 10.2 and 9.65 kilobases. This assembly was generated as part of the Darwin Tree of Life project, which produces reference genomes for eukaryotic species found in Britain and Ireland.

## Species taxonomy

Eukaryota; Opisthokonta; Metazoa; Eumetazoa; Bilateria; Protostomia; Ecdysozoa; Panarthropoda; Arthropoda; Mandibulata; Myriapoda; Diplopoda; Helminthomorpha; Julida; Blaniulidae; *Choneiulus*; *Choneiulus palmatus* (Nĕmec, 1895) (NCBI:txid1008849)

## Background

*Choneiulus palmatus* is found in many countries in western, central and northern Europe ( Kime & Enghoff, 2017), its most southerly records being in southern France and most northerly in northern Sweden. Its native distribution appears to the Atlantic zone of northwest Europe, with its northern and eastern records being synanthropic, and it is introduced to Madeira, the Azores, North America and Tasmania ( Kime, 1999; Kime & Enghoff, 2017; Shelley & Enghoff, 2004). It is widespread but uncommon in Britain and Ireland.

British records, summarised from Lee (2006), are strongly associated with buildings and urban sites, and synanthropic occurrence is strongest in northern England and in Scotland. In more southerly parts of Britain and Ireland it is often likewise synanthropic, found in buildings, waste ground, churchyards and cultivation, but also occurs in leaf litter and under bark in deciduous woodland. Native Continental records are usually in the soil or above ground under the bark or in dead wood of old trees, often on basic and calcareous bedrock ( Kime, 2004). Its European habitats include caves, and nests of ants and moles ( Kime & Enghoff, 2017). In the British and Irish Isles, adults have been collected from February to June and from September to November. Its altitudinal range is to 1 230 m in the Alps. Like other synanthropic Blaniulidae, it may occasionally be an agricultural pest ( Enghoff, 1984).

*Choneiulus palmatus* is the only British or Irish species of the genus, which has four other species in the Mediterranean parts of Europe and North Africa and the Canary Islands ( Enghoff, 1984). It is greyish-brown, with dark spots along its sides, reaching a length to 15 mm in males and 12 mm in females, with up to 58 body rings. It is distinguished from other blaniulids in the British and Irish Isles by its arrangement of ocelli in a single row that parallels the ventral margin of the head capsule, relatively numerous (14 to 20), long setae on the dorsal and dorsolateral margins of the metazonites of its body rings, and by its posterior gonopods terminating in a funnel with fringed margins ( Blower, 1985). Postembryonic development is described from a population in Finland over multiple years ( Peitsalmi & Pajunen, 1996).

Few whole genome sequences for millipedes have been generated: these include *Cylindroiulus punctatus* (GCA_965125795.1) ( Edgecombe *et al.*, 2025), *Nanogona polydesmoides* ( Owen *et al.*, 2025), *Trigoniulus corallinus* (GCA_013389805.1) ( Kenny *et al.*, 2015), *Helicorthomorpha holstii* (GCA_013389785.1) ( Qu *et al.*, 2020), and *Glomeris maerens* (GCA_023279145.1) ( So *et al.*, 2022). The MetaInvert database at Senckenberg Görlitz and the LOEWE Centre for Translational Biodiversity Genomics provides draft genome sequences for many soil invertebrates, among them 23 Diplopoda, including a contig-level assembly for *Choneiulus palmatus* (GCA_034695065.1) ( Collins *et al.*, 2023).

**Figure 1.**
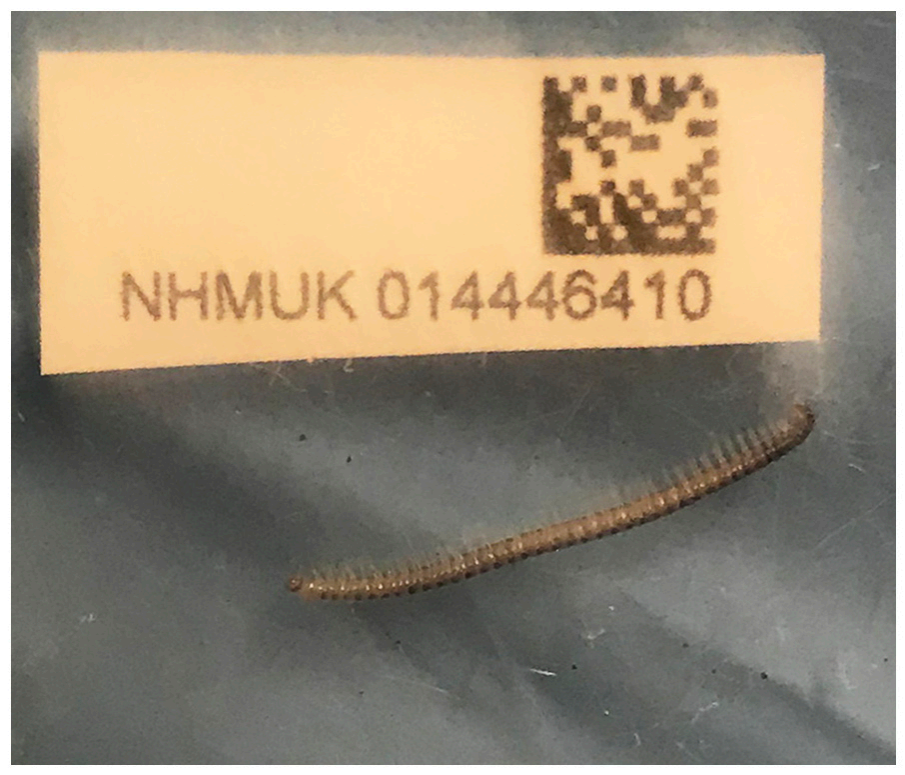
Photograph of the *Choneiulus palmatus* (qdChoPalm2) specimen used for genome sequencing.

Here we present a chromosome-level genome sequence for *Choneiulus palmatus*, based on one adult female specimen from Abingdon, Oxfordshire, England ( Figure 1).

## Methods

### Sample acquisition and DNA barcoding

Adult *Choneiulus palmatus* were collected from Parsonage Moor, Abingdon, Oxfordshire (historic vice-county Berkshire), England, UK (latitude 51.69, longitude –1.33) on 2021-11-11 by handpicking from deep leaf litter at the edge of deciduous woodland. The specimens were collected and identified by Steve Gregory and then preserved in 70% ethanol. One specimen (specimen ID NHMUK014446410, ToLID qdChoPalm2) was used for PacBio HiFi sequencing and another (specimen ID NHMUK014446415, ToLID qdChoPalm1) was used for Hi-C sequencing. Both specimens were adult female millipedes. For the Darwin Tree of Life sampling and metadata approach, refer to Lawniczak *et al.* (2022).

The initial identification was verified by an additional DNA barcoding process according to the framework developed by Twyford *et al.* (2024). A small sample was dissected from the specimen and stored in ethanol, while the remaining parts were shipped on dry ice to the Wellcome Sanger Institute (WSI) (see the protocol). The tissue was lysed, the COI marker region was amplified by PCR, and amplicons were sequenced and compared to the BOLD database, confirming the species identification ( Crowley *et al.*, 2023). Following whole genome sequence generation, the relevant DNA barcode region was also used alongside the initial barcoding data for sample tracking at the WSI ( Twyford *et al.*, 2024). The standard operating procedures for Darwin Tree of Life barcoding are available on protocols.io.

### Nucleic acid extraction

Protocols for high molecular weight (HMW) DNA extraction developed at the Wellcome Sanger Institute (WSI) Tree of Life Core Laboratory are available on protocols.io ( Howard *et al.*, 2025). The qdChoPalm2 sample was weighed and triaged to determine the appropriate extraction protocol. Tissue from the mid body was homogenised by powermashing using a PowerMasher II tissue disruptor. HMW DNA was extracted using the Automated MagAttract v2 protocol. We used centrifuge-mediated fragmentation to produce DNA fragments in the 8–10 kb range, following the Covaris g-TUBE protocol for ultra-low input (ULI). Sheared DNA was purified by automated SPRI (solid-phase reversible immobilisation). The concentration of the sheared and purified DNA was assessed using a Nanodrop spectrophotometer and Qubit Fluorometer using the Qubit dsDNA High Sensitivity Assay kit. Fragment size distribution was evaluated by running the sample on the FemtoPulse system. For this sample, the final post-shearing DNA had a Qubit concentration of 0.988 ng/μL and a yield of 385.32 ng. The 260/280 spectrophotometric ratio was 1.53, and the 260/230 ratio was 3.1.

### PacBio HiFi library preparation and sequencing

Library preparation and sequencing were performed at the WSI Scientific Operations core. Prior to library preparation, the DNA was fragmented to ~10 kb. Ultra-low-input (ULI) libraries were prepared using the PacBio SMRTbell® Express Template Prep Kit 2.0 and gDNA Sample Amplification Kit. Samples were normalised to 20 ng DNA. Single-strand overhang removal, DNA damage repair, and end-repair/A-tailing were performed according to the manufacturer’s instructions, followed by adapter ligation. A 0.85× pre-PCR clean-up was carried out with Promega ProNex beads.

The DNA was evenly divided into two aliquots for dual PCR (reactions A and B), both following the manufacturer’s protocol. A 0.85× post-PCR clean-up was performed with ProNex beads. DNA concentration was measured using a Qubit Fluorometer v4.0 (Thermo Fisher Scientific) with the Qubit HS Assay Kit, and fragment size was assessed on an Agilent Femto Pulse Automated Pulsed Field CE Instrument (Agilent Technologies) using the gDNA 55 kb BAC analysis kit. PCR reactions A and B were then pooled, ensuring a total mass of ≥500 ng in 47.4 μl.

The pooled sample underwent another round of DNA damage repair, end-repair/A-tailing, and hairpin adapter ligation. A 1× clean-up was performed with ProNex beads, followed by DNA quantification using the Qubit and fragment size analysis using the Agilent Femto Pulse. Size selection was performed on the Sage Sciences PippinHT system, with target fragment size determined by Femto Pulse analysis (typically 4–9 kb). Size-selected libraries were cleaned with 1.0× ProNex beads and normalised to 2 nM before sequencing.

The sample was sequenced on a Revio instrument (Pacific Biosciences). The prepared library was normalised to 2 nM, and 15 μL was used for making complexes. Primers were annealed and polymerases bound to generate circularised complexes, following the manufacturer’s instructions. Complexes were purified using 1.2X SMRTbell beads, then diluted to the Revio loading concentration (200–300 pM) and spiked with a Revio sequencing internal control. The sample was sequenced on a Revio 25M SMRT cell. The SMRT Link software (Pacific Biosciences), a web-based workflow manager, was used to configure and monitor the run and to carry out primary and secondary data analysis.

### Hi-C

***Sample preparation and crosslinking***

The Hi-C sample was prepared from 20–50 mg of frozen tissue from the mid-body of the qdChoPalm1 sample using the Arima-HiC v2 kit (Arima Genomics). Following the manufacturer’s instructions, tissue was fixed and DNA crosslinked using TC buffer to a final formaldehyde concentration of 2%. The tissue was homogenised using the Diagnocine Power Masher-II. Crosslinked DNA was digested with a restriction enzyme master mix, biotinylated, and ligated. Clean-up was performed with SPRISelect beads before library preparation. DNA concentration was measured with the Qubit Fluorometer (Thermo Fisher Scientific) and Qubit HS Assay Kit. The biotinylation percentage was estimated using the Arima-HiC v2 QC beads.

***Hi-C library preparation and sequencing***

Biotinylated DNA constructs were fragmented using a Covaris E220 sonicator and size selected to 400–600 bp using SPRISelect beads. DNA was enriched with Arima-HiC v2 kit Enrichment beads. End repair, A-tailing, and adapter ligation were carried out with the NEBNext Ultra II DNA Library Prep Kit (New England Biolabs), following a modified protocol where library preparation occurs while DNA remains bound to the Enrichment beads. Library amplification was performed using KAPA HiFi HotStart mix and a custom Unique Dual Index (UDI) barcode set (Integrated DNA Technologies). Depending on sample concentration and biotinylation percentage determined at the crosslinking stage, libraries were amplified with 10 to 16 PCR cycles. Post-PCR clean-up was performed with SPRISelect beads. Libraries were quantified using the AccuClear Ultra High Sensitivity dsDNA Standards Assay Kit (Biotium) and a FLUOstar Omega plate reader (BMG Labtech).

Prior to sequencing, libraries were normalised to 10 ng/μL. Normalised libraries were quantified again and equimolar and/or weighted 2.8 nM pools. Pool concentrations were checked using the Agilent 4200 TapeStation (Agilent) with High Sensitivity D500 reagents before sequencing. Sequencing was performed using paired-end 150 bp reads on the Illumina NovaSeq 6000.

### Genome assembly

Prior to assembly of the PacBio HiFi reads, a database of *k*-mer counts ( *k* = 31) was generated from the filtered reads using FastK. GenomeScope2 ( Ranallo-Benavidez *et al.*, 2020) was used to analyse the *k*-mer frequency distributions, providing estimates of genome size, heterozygosity, and repeat content.

The HiFi reads were assembled using Hifiasm ( Cheng *et al.*, 2021) with the --primary option. Haplotypic duplications were identified and removed using purge_dups ( Guan *et al.*, 2020). The Hi-C reads ( Rao *et al.*, 2014) were mapped to the primary contigs using bwa-mem2 ( Vasimuddin *et al.*, 2019), and the contigs were scaffolded in YaHS ( Zhou *et al.*, 2023) with the --break option for handling potential misassemblies. The scaffolded assemblies were evaluated using Gfastats ( Formenti *et al.*, 2022), BUSCO ( Manni *et al.*, 2021) and MERQURY.FK ( Rhie *et al.*, 2020).

The mitochondrial genome was assembled using OATK ( Zhou *et al.*, 2025) and uses these annotations to select the final mitochondrial contig and to ensure the general quality of the sequence.

### Assembly curation

The assembly was decontaminated using the Assembly Screen for Cobionts and Contaminants ( ASCC) pipeline. TreeVal was used to generate the flat files and maps for use in curation. Manual curation was conducted primarily in PretextView and HiGlass ( Kerpedjiev *et al.*, 2018). Scaffolds were visually inspected and corrected as described by Howe *et al.* (2021). Manual corrections included 56 breaks and 71 joins. The curation process is documented at https://gitlab.com/wtsi-grit/rapid-curation. PretextSnapshot was used to generate a Hi-C contact map of the final assembly.

### Assembly quality assessment

The Merqury.FK tool ( Rhie *et al.*, 2020) was run in a Singularity container ( Kurtzer *et al.*, 2017) to evaluate *k*-mer completeness and assembly quality for the primary and alternate haplotypes using the *k*-mer databases ( *k* = 31) computed prior to genome assembly. The analysis outputs included assembly QV scores and completeness statistics.

The genome was analysed using the BlobToolKit pipeline, a Nextflow implementation of the earlier Snakemake version ( Challis *et al.*, 2020). The pipeline aligns PacBio reads using minimap2 ( Li, 2018) and SAMtools ( Danecek *et al.*, 2021) to generate coverage tracks. It runs BUSCO ( Manni *et al.*, 2021) using lineages identified from the NCBI Taxonomy ( Schoch *et al.*, 2020). For the three domain-level lineages, BUSCO genes are aligned to the UniProt Reference Proteomes database ( Bateman *et al.*, 2023) using DIAMOND blastp ( Buchfink *et al.*, 2021). The genome is divided into chunks based on the density of BUSCO genes from the closest taxonomic lineage, and each chunk is aligned to the UniProt Reference Proteomes database with DIAMOND blastx. Sequences without hits are chunked using seqtk and aligned to the NT database with blastn ( Altschul *et al.*, 1990). The BlobToolKit suite consolidates all outputs into a blobdir for visualisation. The BlobToolKit pipeline was developed using nf-core tooling ( Ewels *et al.*, 2020) and MultiQC ( Ewels *et al.*, 2016), with containerisation through Docker ( Merkel, 2014) and Singularity ( Kurtzer *et al.*, 2017).

## Genome sequence report

### Sequence data

**Figure 2.**
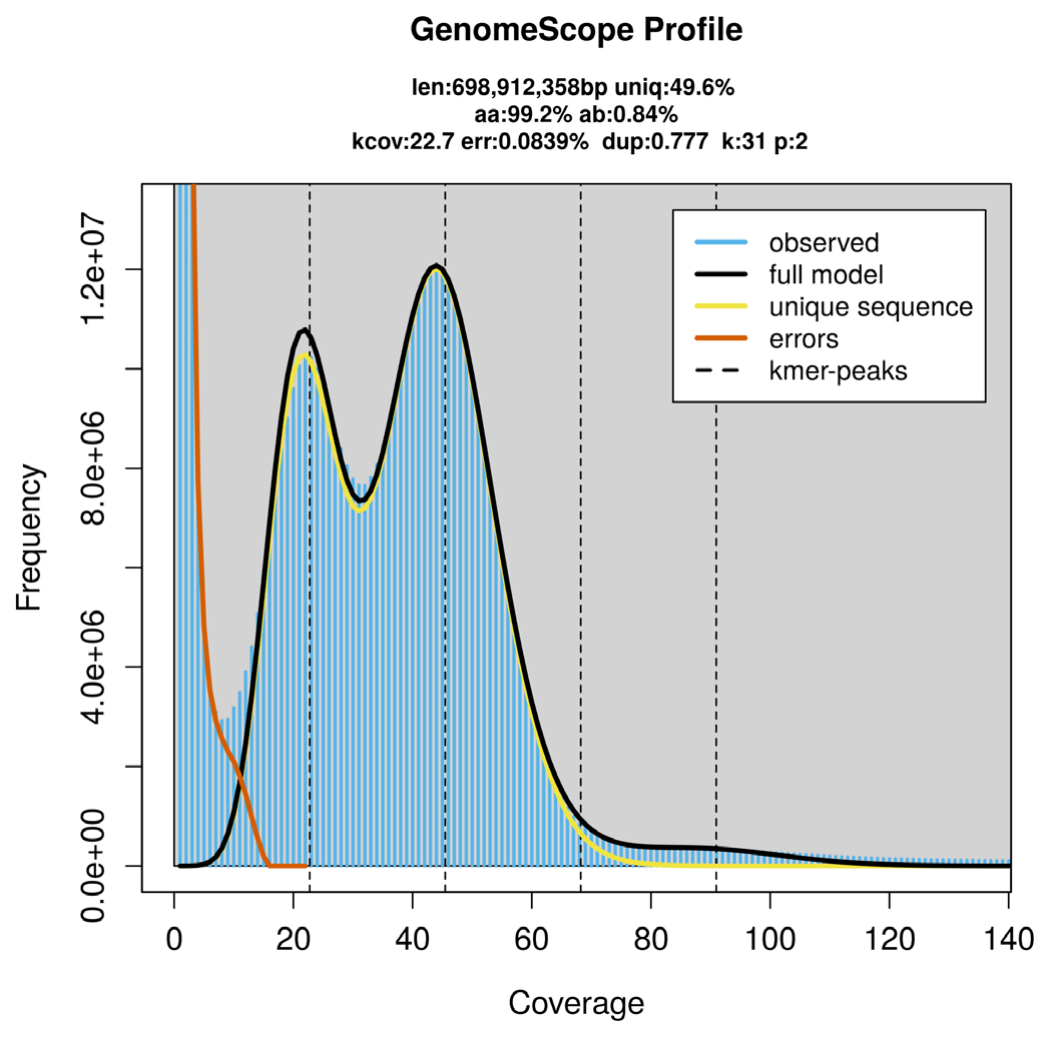
Frequency distribution of *k*-mers generated using GenomeScope2. The plot shows observed and modelled *k*-mer spectra, providing estimates of genome size, heterozygosity, and repeat content based on unassembled sequencing reads.

PacBio sequencing of the *Choneiulus palmatus* specimen generated 33.82 Gb (gigabases) from 4.69 million reads, which were used to assemble the genome. GenomeScope2.0 analysis estimated the haploid genome size at 698.91 Mb, with a heterozygosity of 0.84% and repeat content of 50.63% ( Figure 2). These estimates guided expectations for the assembly. Based on the estimated genome size, the sequencing data provided approximately 45× coverage. Hi-C sequencing produced 109.50 Gb from 725.14 million reads, which were used to scaffold the assembly. Table 1 summarises the specimen and sequencing details.

**Table 1.** Specimen and sequencing data for BioProject PRJEB81632.

Platform	PacBio HiFi	Hi-C
**ToLID**	qdChoPalm2	qdChoPalm1
**Specimen ID**	NHMUK014446410	NHMUK014446415
**BioSample (source individual)**	SAMEA110034692	SAMEA14448368
**BioSample (tissue)**	SAMEA14448638	SAMEA14448637
**Tissue**	mid body	mid body
**Instrument**	Revio	Illumina NovaSeq 6000
**Run accessions**	ERR13900458	ERR13907237
**Read count total**	4.69 million	725.14 million
**Base count total**	33.82 Gb	109.50 Gb

### Assembly statistics

The primary haplotype was assembled, and contigs corresponding to an alternate haplotype were also deposited in INSDC databases. The final assembly has a total length of 626.52 Mb in 182 scaffolds, with 814 gaps, and a scaffold N50 of 42.36 Mb ( Table 2).

**Table 2.** Genome assembly statistics.

**Assembly name**	qdChoPalm2.1
**Assembly accession**	GCA_965278725.1
**Alternate haplotype accession**	GCA_965278825.1
**Assembly level**	chromosome
**Span (Mb)**	626.52
**Number of chromosomes**	14
**Number of contigs**	996
**Contig N50**	1.19 Mb
**Number of scaffolds**	182
**Scaffold N50**	42.36 Mb
**Sex chromosomes**	not identified
**Organelles**	Mitochondrial sequences: 10.2 and 9.65 kb

**Figure 3.**
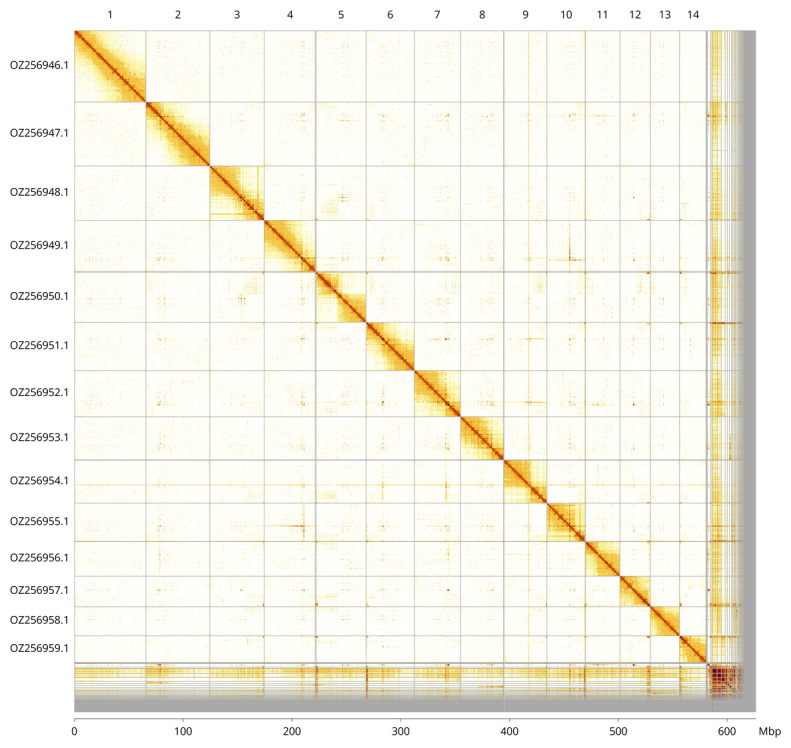
Hi-C contact map of the *Choneiulus palmatus* genome assembly. Assembled chromosomes are shown in order of size and labelled along the axes, with a megabase scale shown below. The plot was generated using PretextSnapshot.

Most of the assembly sequence (92.77%) was assigned to 14 chromosomal-level scaffolds. These chromosome-level scaffolds, confirmed by Hi-C data, are named according to size ( Figure 3; Table 3). The exact order and orientation of the contigs on chromosome 3 (26 200–32 500 Kbp) are unknown.

**Table 3.** Chromosomal pseudomolecules in the primary genome assembly of *Choneiulus palmatus* qdChoPalm2.

INSDC accession	Molecule	Length (Mb)	GC%
OZ256946.1	1	65.86	33.50
OZ256947.1	2	58.76	33.50
OZ256948.1	3	49.86	33.50
OZ256949.1	4	47.82	33.50
OZ256950.1	5	45.87	34
OZ256951.1	6	44.37	33
OZ256952.1	7	42.36	33.50
OZ256953.1	8	40.07	33
OZ256954.1	9	39.43	34
OZ256955.1	10	35.34	33
OZ256956.1	11	31.71	33
OZ256957.1	12	28.02	34
OZ256958.1	13	26.79	32.50
OZ256959.1	14	24.95	34.50

The mitochondrial genome was also assembled. This sequence is included as a contig in the multifasta file of the genome submission and as a standalone record.

**Figure 4.**
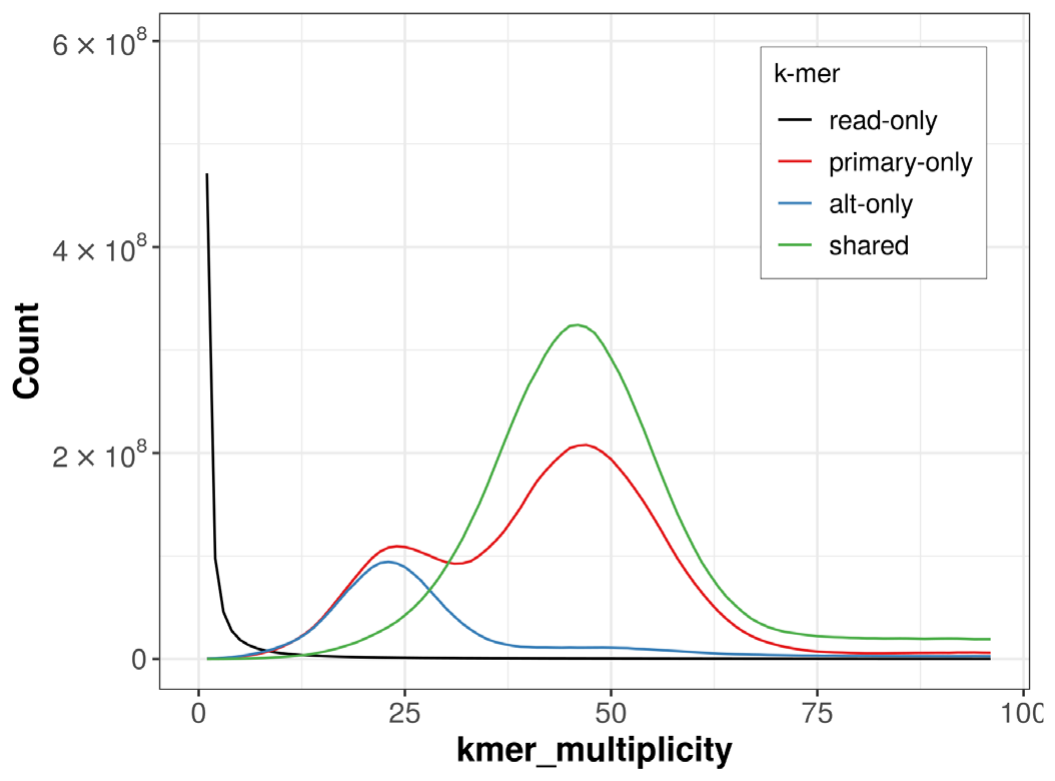
Evaluation of *k*-mer completeness using MerquryFK. This plot illustrates the recovery of *k*-mers from the original read data in the final assemblies. The horizontal axis represents *k*-mer multiplicity, and the vertical axis shows the number of *k*-mers. The black curve represents *k*-mers that appear in the reads but are not assembled. The green curve corresponds to *k*-mers shared by both haplotypes, and the red and blue curves show *k*-mers found only in one of the haplotypes.

The combined primary and alternate assemblies achieve an estimated QV of 58.4. The *k*-mer completeness is 82.96% for the primary assembly, 60.70% for the alternate haplotype, and 99.05% for the combined assemblies ( Figure 4).

**Figure 5.**
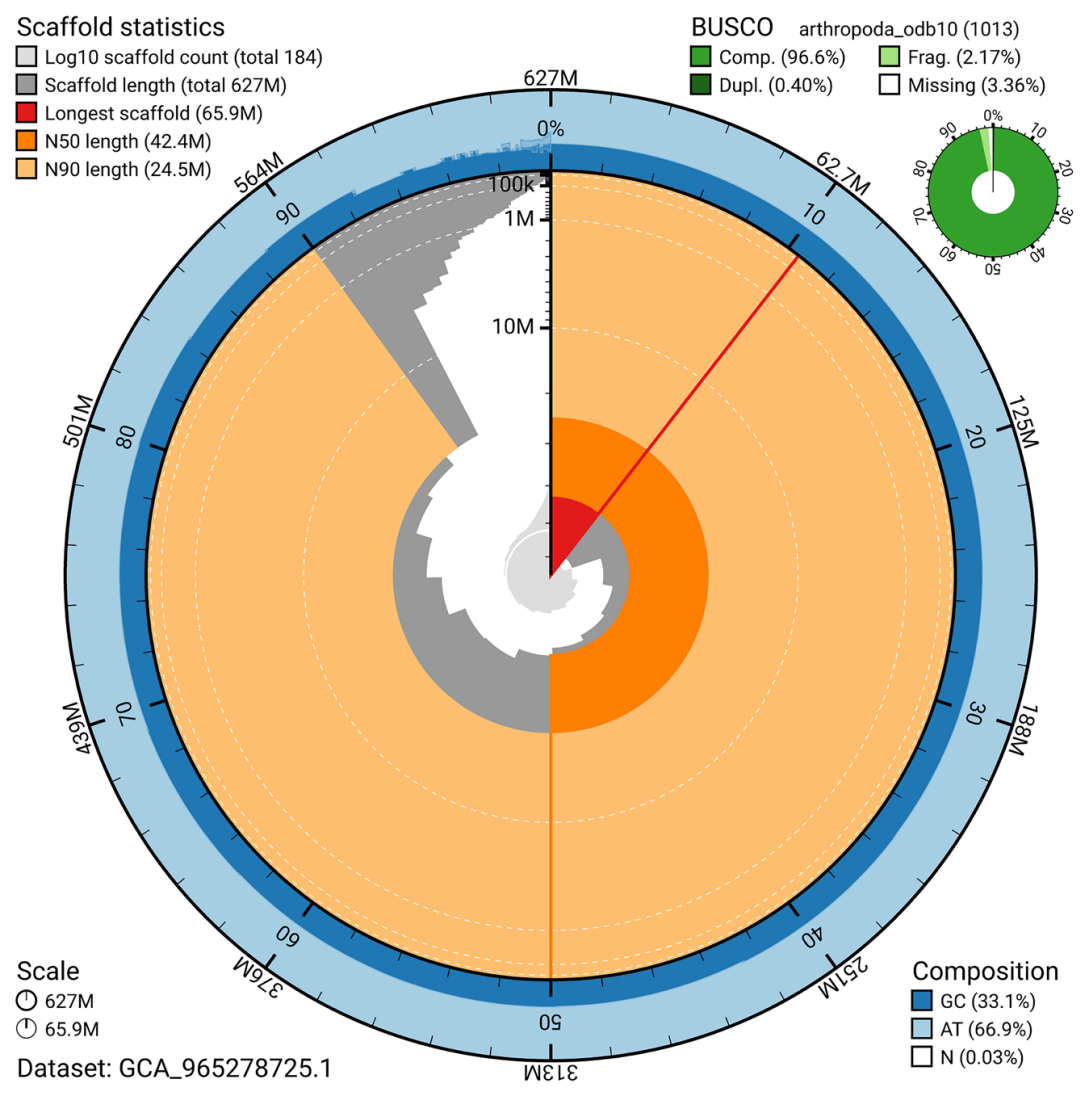
Assembly metrics for qdChoPalm2.1. The BlobToolKit snail plot provides an overview of assembly metrics and BUSCO gene completeness. The circumference represents the length of the whole genome sequence, and the main plot is divided into 1 000 bins around the circumference. The outermost blue tracks display the distribution of GC, AT, and N percentages across the bins. Scaffolds are arranged clockwise from longest to shortest and are depicted in dark grey. The longest scaffold is indicated by the red arc, and the deeper orange and pale orange arcs represent the N50 and N90 lengths. A light grey spiral at the centre shows the cumulative scaffold count on a logarithmic scale. A summary of complete, fragmented, duplicated, and missing BUSCO genes in the arthropoda_odb10 set is presented at the top right. An interactive version of this figure can be accessed on the BlobToolKit viewer.

**Figure 6.**
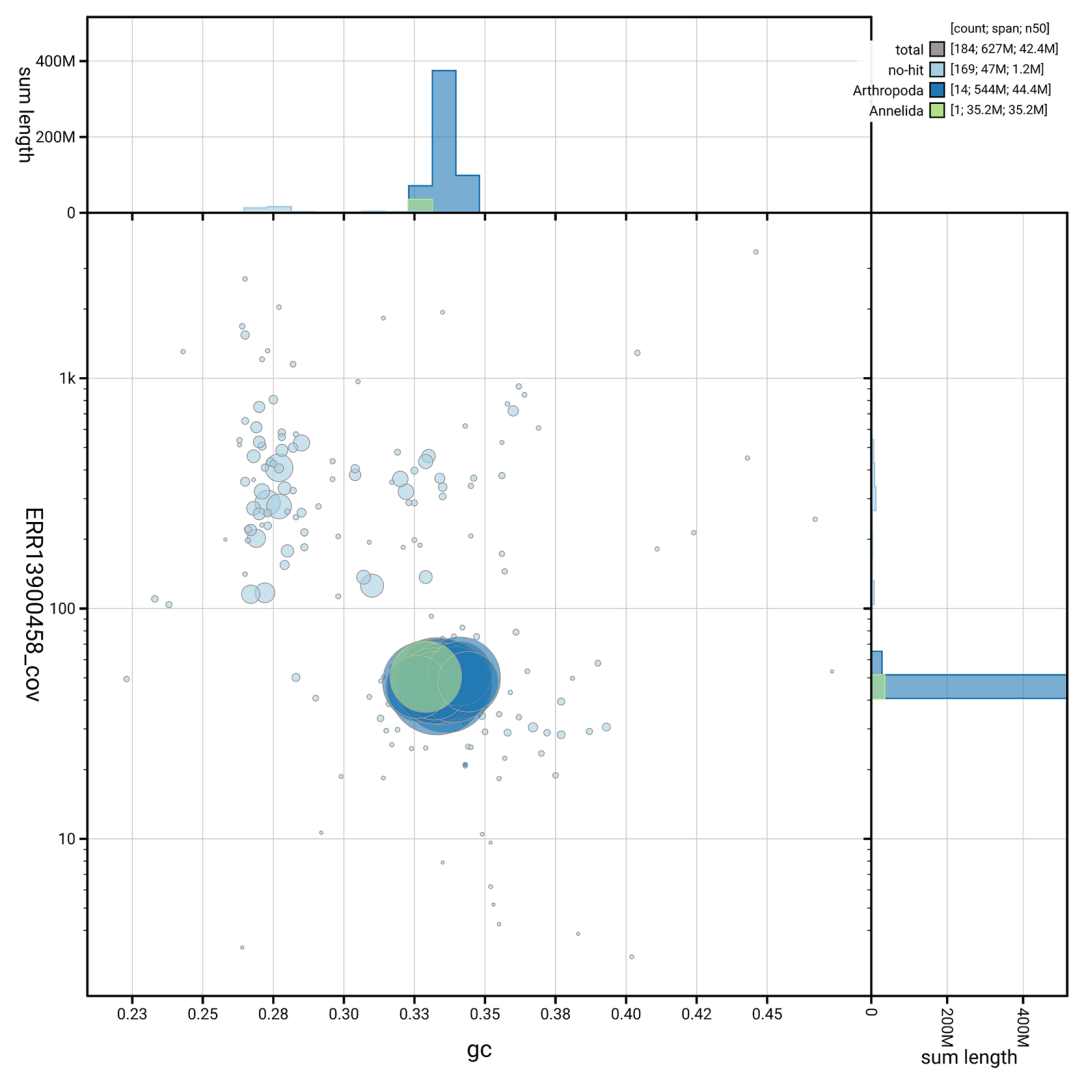
BlobToolKit GC-coverage plot for qdChoPalm2.1. Blob plot showing sequence coverage (vertical axis) and GC content (horizontal axis). The circles represent scaffolds, with the size proportional to scaffold length and the colour representing phylum membership. The histograms along the axes display the total length of sequences distributed across different levels of coverage and GC content. An interactive version of this figure is available on the BlobToolKit viewer.

BUSCO v.5.7.1 analysis using the arthropoda_odb10 reference set ( *n* = 1 013) identified 96.6% of the expected gene set (single = 96.2%, duplicated = 0.4%). The snail plot in Figure 5 summarises the scaffold length distribution and other assembly statistics for the primary assembly. The blob plot in Figure 6 shows the distribution of scaffolds by GC proportion and coverage.

Table 4 lists the assembly metric benchmarks adapted from Rhie *et al.* (2021) the Earth BioGenome Project Report on Assembly Standards September 2024. The EBP metric, calculated for the primary assembly, is **6.C.Q59**, meeting the recommended reference standard.

**Table 4.** Earth Biogenome Project summary metrics for the *Choneiulus palmatus* assembly.

Measure	Value	Benchmark
EBP summary (primary)	6.C.Q59	6.C.Q40
Contig N50 length	1.19 Mb	≥ 1 Mb
Scaffold N50 length	42.36 Mb	= chromosome N50
Consensus quality (QV)	Primary: 59.2; alternate: 57.9; combined: 58.4	≥ 40
*k*-mer completeness	Primary: 82.96%; alternate: 60.70%; combined: 99.05%	≥ 95%
BUSCO	C:96.6% [S:96.2%; D:0.4%]; F:2.2%; M:1.2%; n:1 013	S > 90%; D < 5%
Percentage of assembly assigned to chromosomes	92.77%	≥ 90%

### Wellcome Sanger Institute – Legal and Governance

The materials that have contributed to this genome note have been supplied by a Darwin Tree of Life Partner. The submission of materials by a Darwin Tree of Life Partner is subject to the **‘Darwin Tree of Life Project Sampling Code of Practice’**, which can be found in full on the Darwin Tree of Life website. By agreeing with and signing up to the Sampling Code of Practice, the Darwin Tree of Life Partner agrees they will meet the legal and ethical requirements and standards set out within this document in respect of all samples acquired for, and supplied to, the Darwin Tree of Life Project. Further, the Wellcome Sanger Institute employs a process whereby due diligence is carried out proportionate to the nature of the materials themselves, and the circumstances under which they have been/are to be collected and provided for use. The purpose of this is to address and mitigate any potential legal and/or ethical implications of receipt and use of the materials as part of the research project, and to ensure that in doing so we align with best practice wherever possible. The overarching areas of consideration are:

- Ethical review of provenance and sourcing of the material
- Legality of collection, transfer and use (national and international)

Each transfer of samples is further undertaken according to a Research Collaboration Agreement or Material Transfer Agreement entered into by the Darwin Tree of Life Partner, Genome Research Limited (operating as the Wellcome Sanger Institute), and in some circumstances, other Darwin Tree of Life collaborators.

## Data Availability

European Nucleotide Archive: Choneiulus palmatus. Accession number PRJEB81632. The genome sequence is released openly for reuse. The *Choneiulus palmatus* genome sequencing initiative is part of the Darwin Tree of Life Project (PRJEB40665) and the Sanger Institute Tree of Life Programme (PRJEB43745). All raw sequence data and the assembly have been deposited in INSDC databases. The genome will be annotated using available RNA-Seq data and presented through the Ensembl pipeline at the European Bioinformatics Institute. Raw data and assembly accession identifiers are reported in Table 1 and Table 2. Production code used in genome assembly at the WSI Tree of Life is available at https://github.com/sanger-tol. Table 5 lists software versions used in this study.

## Author information

Contributors are listed at the following links:

- Members of the Natural History Museum Genome Acquisition Lab
- Members of the Darwin Tree of Life Barcoding collective
- Members of the Wellcome Sanger Institute Tree of Life Management, Samples and Laboratory team
- Members of Wellcome Sanger Institute Scientific Operations – Sequencing Operations
- Members of the Wellcome Sanger Institute Tree of Life Core Informatics team
- Members of the Tree of Life Core Informatics collective
- Members of the Darwin Tree of Life Consortium
