# Supplementary material for: The genome sequence of the millipede, Choneiulus palmatus (Nĕmec, 1895) (Julida: Blaniulidae)
Source: Wellcome Open Res. 2025 Sep 15;10:513. [Version 1] doi: 10.12688/wellcomeopenres.24885.1 (PMC12515362; doi:10.12688/wellcomeopenres.24885.1)
Supplement: Peer review history

# Reviewer report: Reviewer response for version 1

**DOI:** 10.21956/wellcomeopenres.27412.r134595

**Published:** 2025-10-11

**Authors:** Maurijn van der Zee

**Affiliations:** 1 Leiden University, Leiden, The Netherlands

Gergory and Edgecombe provide the genome sequence of the millipede *Choneiulus palmatus*. The genome is of high quality. Hi-C data allowed a reliable assembly into chromosomes, and a BUSCO completeness score of 96.6 % is excellent. (the letters S, D, F and M in the line of the BUSCO score in table 4 were not immediately clear to me, but could readily be understood by looking at figure 5).

 I am aware of mitochondrial sequence variation within one individual, but I was wondering why two mitochondrial sequence assemblies of 10.2 and 9.65 kb are present.

 Finally, as always with such genome projects, hardly any biological interpretation of gene content is provided, but that is also not the aim of this project.  The the presented genome sequence is very reliable and will serve as a useful source of information for other researchers.

Are sufficient details of methods and materials provided to allow replication by others?

Yes

Is the rationale for creating the dataset(s) clearly described?

Yes

Are the datasets clearly presented in a useable and accessible format?

Yes

Are the protocols appropriate and is the work technically sound?

Yes

Reviewer Expertise:

evo-devo, comparative genomics

I confirm that I have read this submission and believe that I have an appropriate level of expertise to confirm that it is of an acceptable scientific standard.

# Reviewer report: Reviewer response for version 1

**DOI:** 10.21956/wellcomeopenres.27412.r133217

**Published:** 2025-09-26

**Authors:** Zhi-Hui Su

**Affiliations:** 1 JT Biohistory Research Hall, Takatsuki, Osaka, Japan

This paper presents a chromosome-level genome sequence for *Choneiulus palmatus*, a species of the myriapoda, order Julida, family Blaniulidae. The methods for sample preparation, DNA extraction, library construction, sequencing, and assembly are all described in detail, and the software used for analysis is clearly indicated, making it easy for readers to understand the contents and access further information. The results are clear, and the quality of the assembled sequence data is very high, as shown by the evaluation scores summarized in Table 4.

 Few whole genome sequences for millipedes have been generated as the authors state, thus the genome sequence reported in this paper is expected to contribute significantly to future research on the phylogeny, evolution, and diversity of millipedes.

 I would like the authors to explain in the paper why they chose this species to determine its whole genome sequence, if they have any background or purpose.

 I hope that in the future, the authors will add annotation information to the whole genome sequence data of this myriapod, if possible.

Are sufficient details of methods and materials provided to allow replication by others?

Yes

Is the rationale for creating the dataset(s) clearly described?

Yes

Are the datasets clearly presented in a useable and accessible format?

Yes

Are the protocols appropriate and is the work technically sound?

Yes

Reviewer Expertise:

Evolutionary Biology

I confirm that I have read this submission and believe that I have an appropriate level of expertise to confirm that it is of an acceptable scientific standard.
